# Supplementary material for: Strong SARS-CoV-2 N-Specific CD8+ T Immunity Induced by Engineered Extracellular Vesicles Associates with Protection from Lethal Infection in Mice
Source: Viruses. 2022 Feb 6;14(2):329. doi: 10.3390/v14020329 (PMC8879411; doi:10.3390/v14020329)
Supplement: Supplementary file 1 [file viruses-14-00329-s001.zip › viruses-1574411-supplementary.pdf]

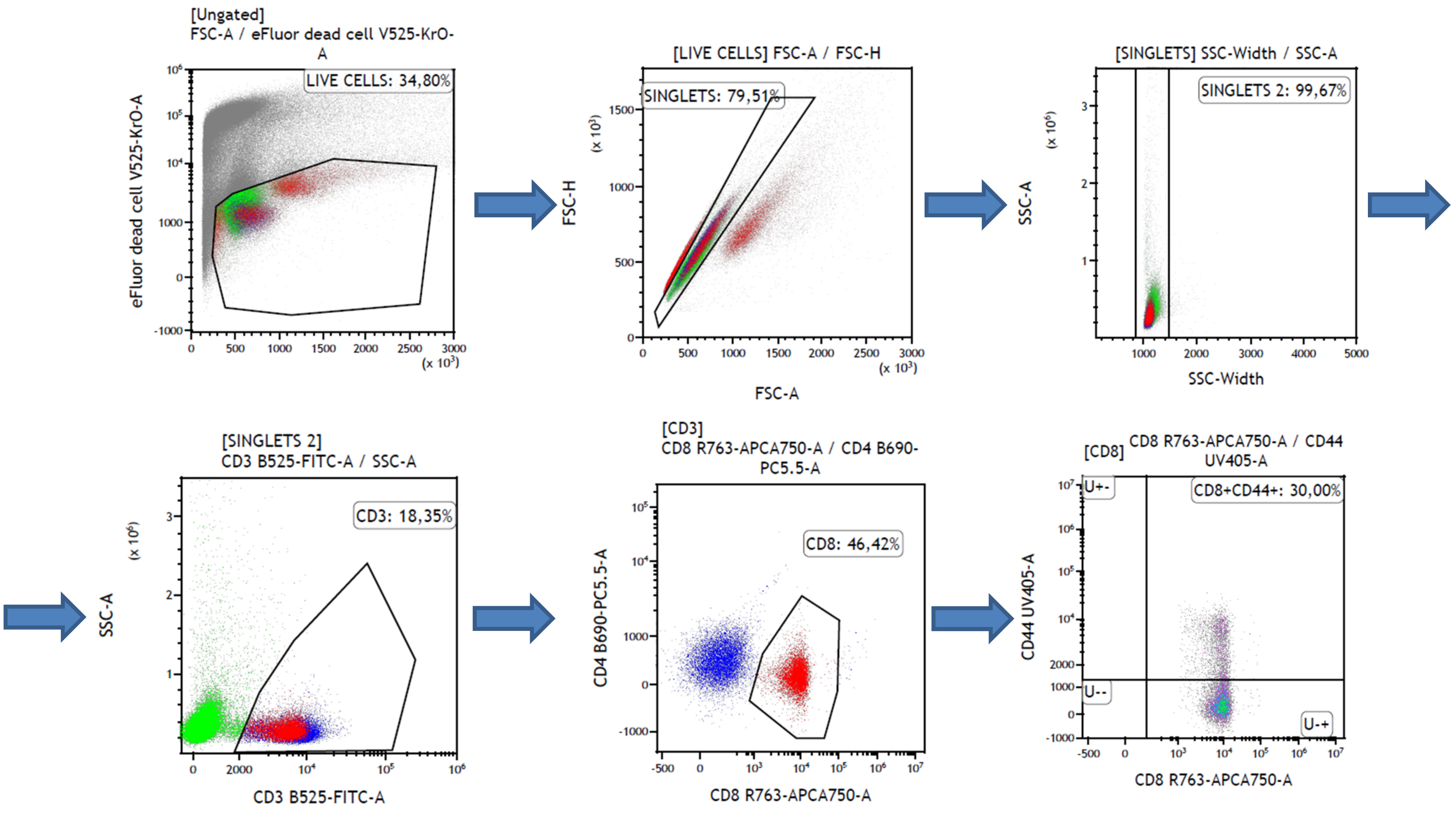

Supplementary Figure S1. Gating strategy carried out in ICS/flow cytometry analysis of splenocytes from injected mice. Shown is the analysis on PMA-treated cells.

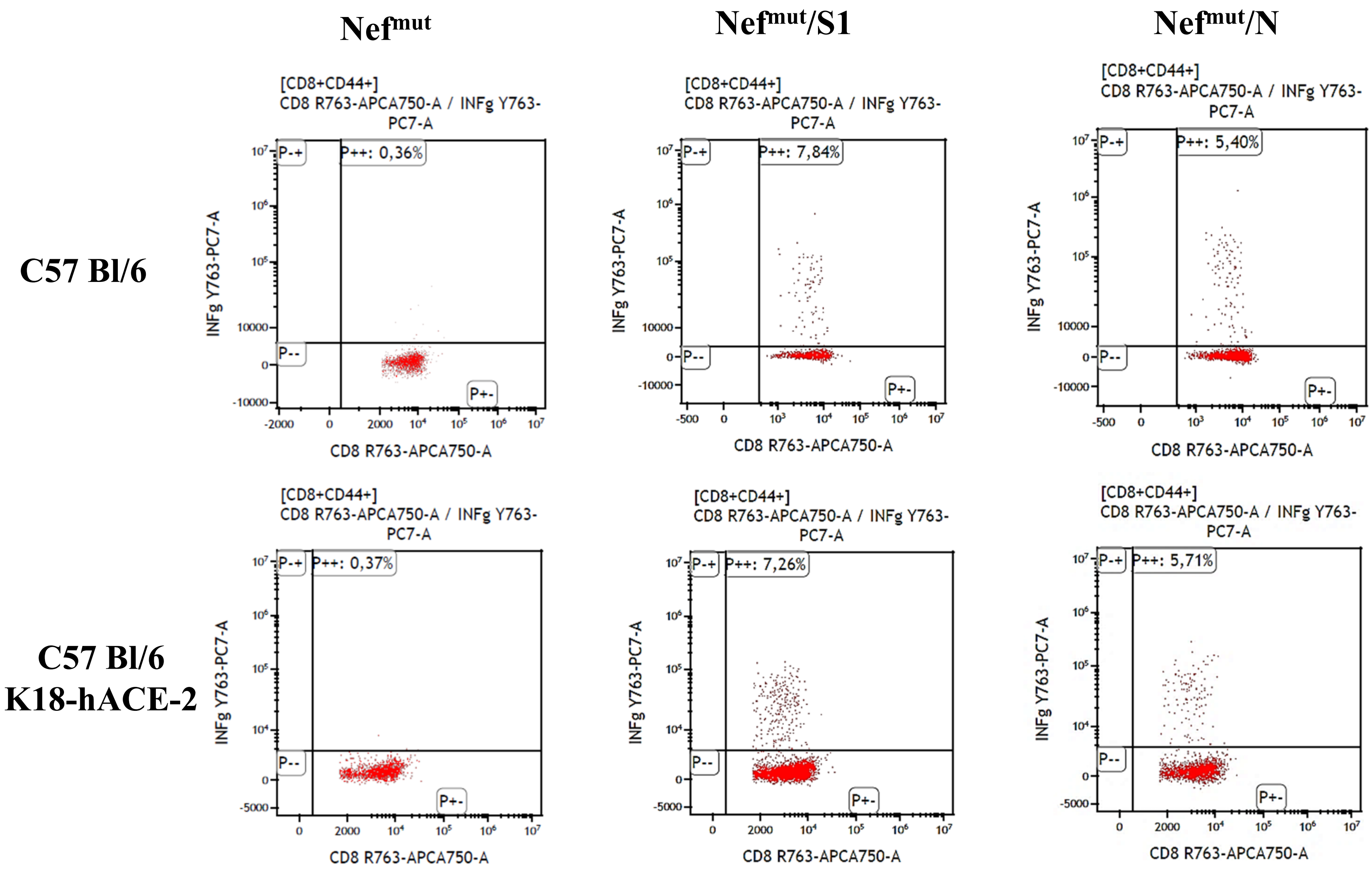

Supplementary Figure S2. ICS/flow cytometry analysis for the expression of IFN-γ in splenocytes from either C57 Bl/ or K18-hACE-2 mice. The mice were injected with vectors expressing either Nef_{mut}/S1, Nef_{mut}/N or, as control, Nef_{mut} alone, and cultivated overnight with SARS-CoV-2 specific peptides. Shown are rough data representative of the results obtained with splenocytes pooled from two mice per condition. Quadrants were set on the basis of cell fluorescence of samples treated with an unrelated peptide.

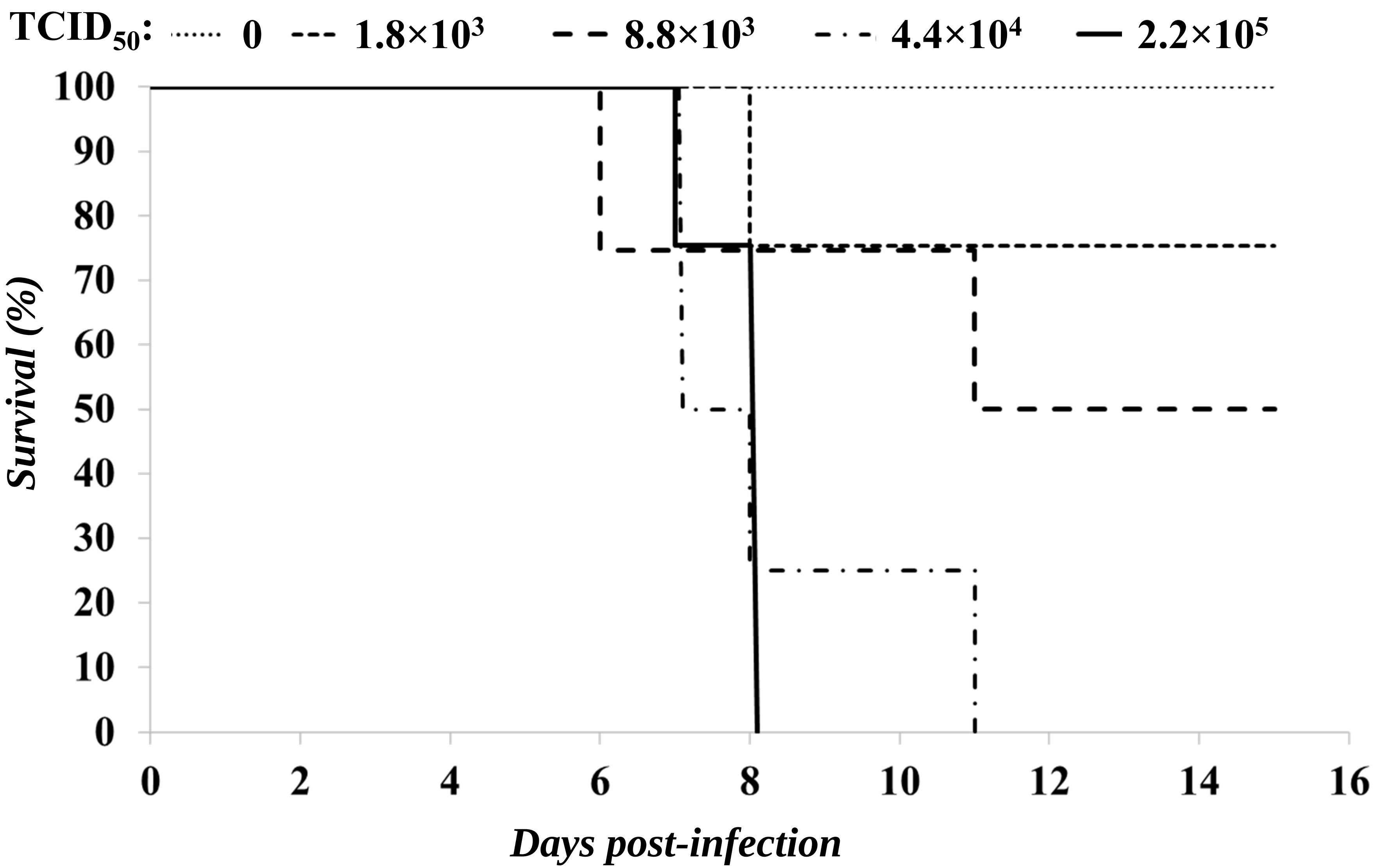

Supplementary Figure S3. Kaplan–Meier survival curve. It was calculated for groups (n=4) of C57 Bl/6 K18-hACE-2 mice infected with 5-fold dilutions of the SARS-CoV-2 preparation.

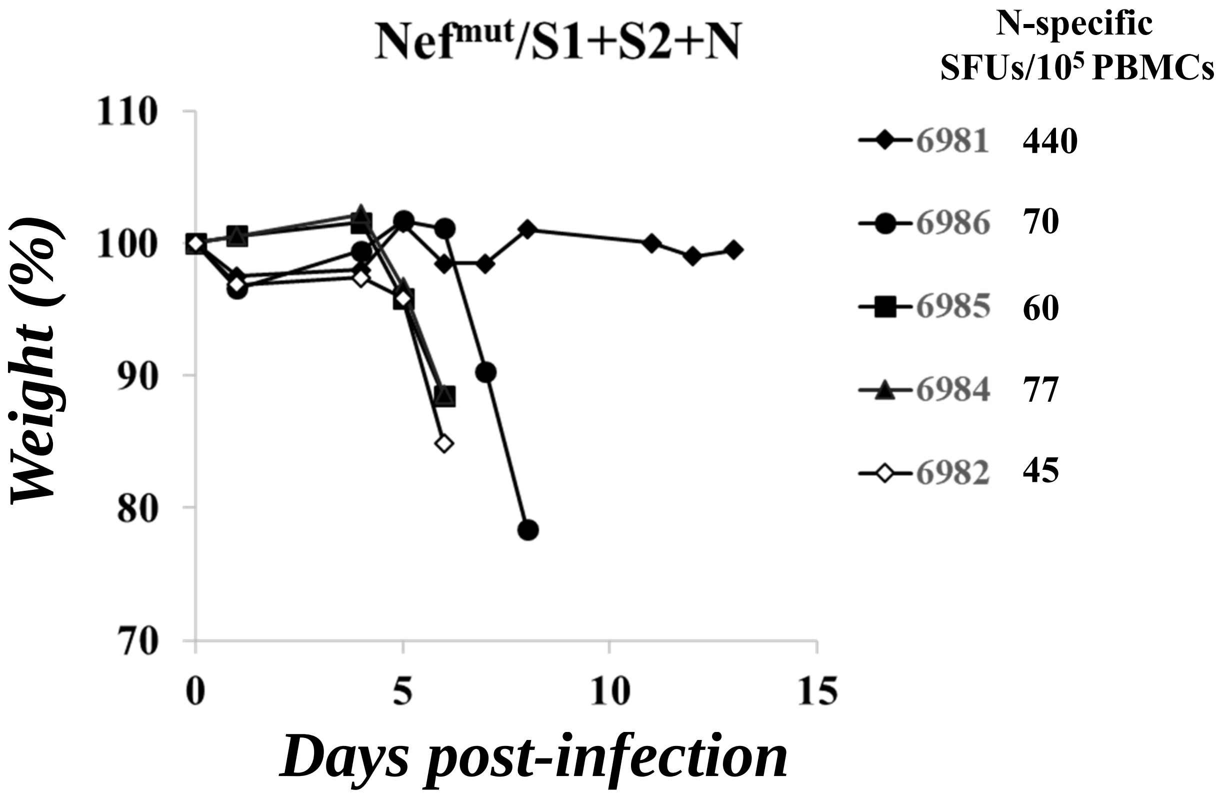

Supplementary Figure S4. Relative weight loss in mice injected with combination vectors. Mice were injected with 10 µg each of Nef^{mut}/S1, Nef^{mut}/S2 and Nef^{mut}/N expressing vectors, and then infected with 5 LD_{50} of SARS-CoV-2. Identification numbers for each mouse are reported on the right together with the counts of SFUs/10^5

PBMCs after incubation with the $N_{219-228}$ peptide. S2-specific SFU counts as measured using a pool of S2 peptides were similar in each mouse.

```
                     Linear Regression    (SFU vs. Survival)
Number of points = 7
           Best-fit  Standard  95% confidence interval
 Parameter    Value     Error      from       to
===========  ========  ========  ========  ========
Slope         28.411     6.115    12.690    44.131
Y intercept   -162.40    69.372   -340.75    15.956
X intercept     5.716
Correlation coefficient (r) = 0.9011.  r squared = 0.8119
Standard deviation of residuals from line (Sy.x) = 60.355
Test: Is the slope significantly different from zero?
The P value is 0.0056, considered very significant.
This result was obtained from the following ANOVA table.
       Source of                   Degrees of   Sum of     Mean
       variation                   freedom    squares    square
===================================  ==========  ========  ========
Linear regression (Model)                   1     78640     78640
Deviations from linearity (Residual)         5     18214    3642.7
-----------------------------------  ----------  --------
Total                                      6     96854
F = 21.588
```

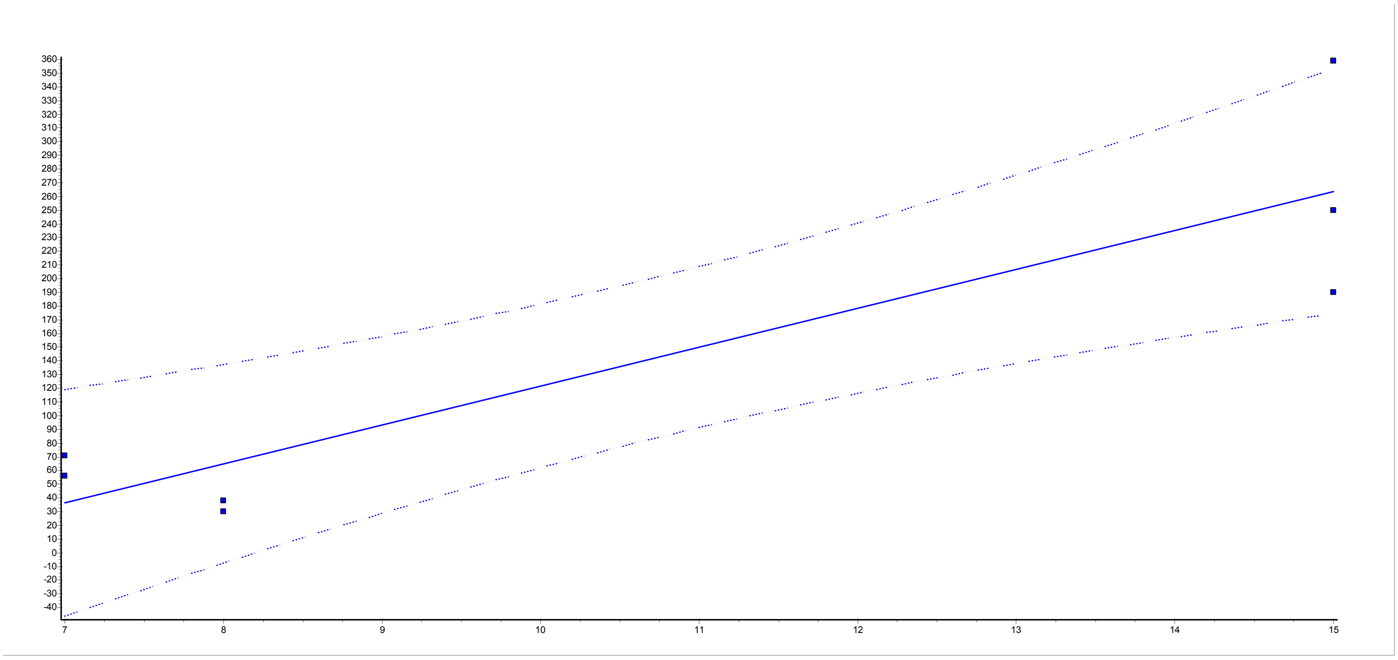

Linear Regression (SFU vs. Weight)

Number of points = 7

Parameter	Best-fit Value	Standard Error	95% confidence interval from	to
Slope	0.06887	0.01014	0.04280	0.09495
Y intercept	83.948	1.870	79.140	88.757
X intercept	-1218.9			

Correlation coefficient (r) = 0.9498. r squared = 0.9022

Standard deviation of residuals from line (Sy.x) = 3.157

Test: Is the slope significantly different from zero?

The P value is 0.0011, considered very significant.

This result was obtained from the following ANOVA table.

Source of variation	Degrees of freedom	Sum of squares	Mean square
Linear regression (Model)	1	459.44	459.44
Deviations from linearity (Residual)	5	49.818	9.964
Total	6	509.25	

F = 46.111

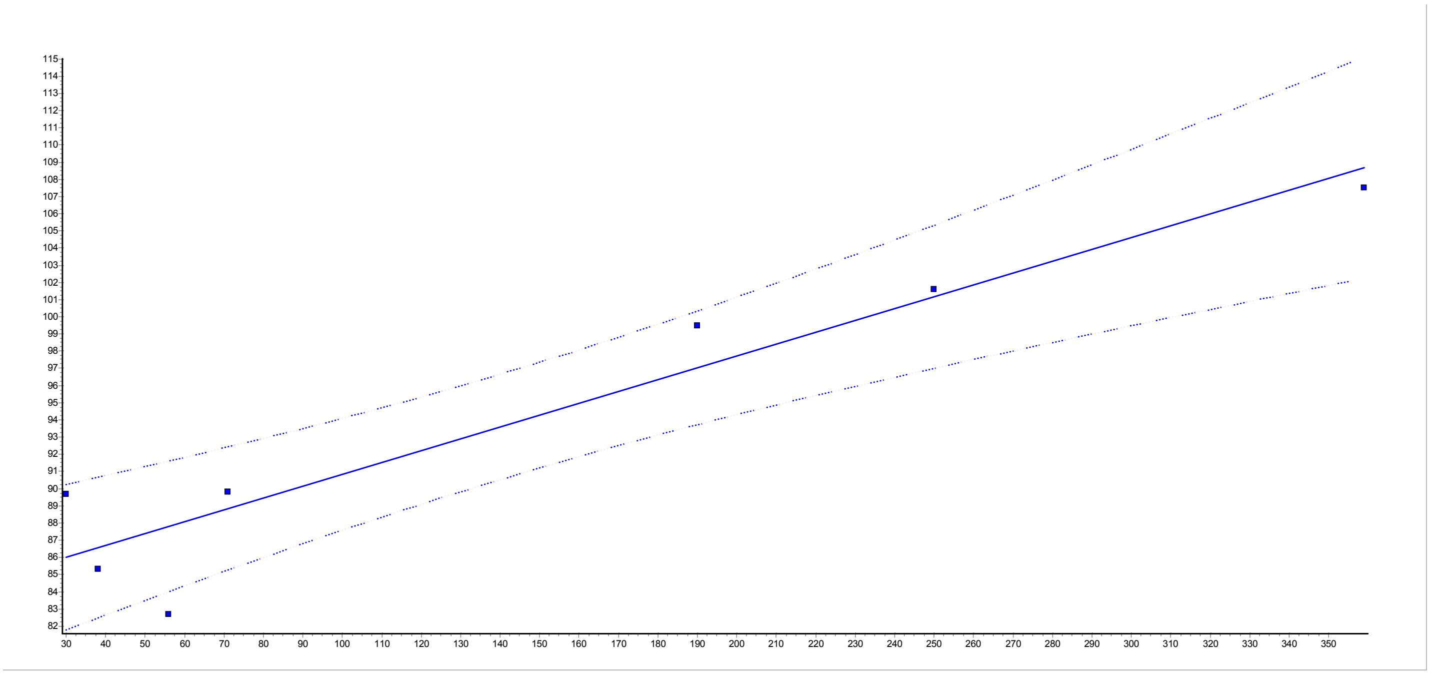

Supplementary Figure S5. Correlation between anti-N SFUs and protection in terms of both mouse relative body weight at day 6/7 post-infection and survival. Regression curves and details of the analysis.

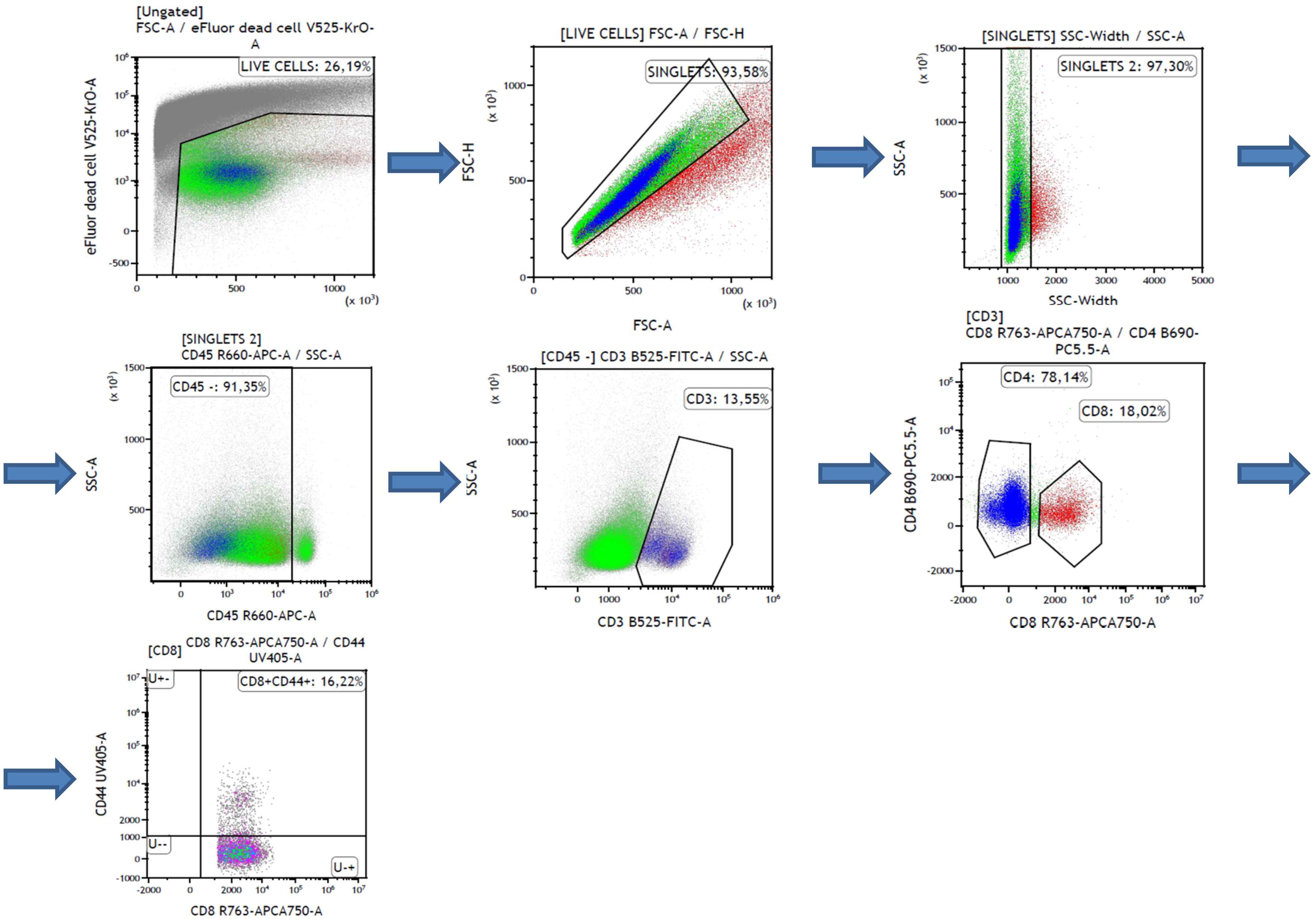

Supplementary Figure S6A. Gating strategy carried out in ICS/flow cytometry analysis of cells pooled from lungs of two mice injected with Nefmut/N expressing vector. Two micrograms of anti-CD45 mAbs (Tonbo-Bioscience CD45.2-APC) diluted in a total of 200 L of 1×PBS were i.v.injected three minutes before sacrifices. The labeling efficiency of leukocytes was 87%.

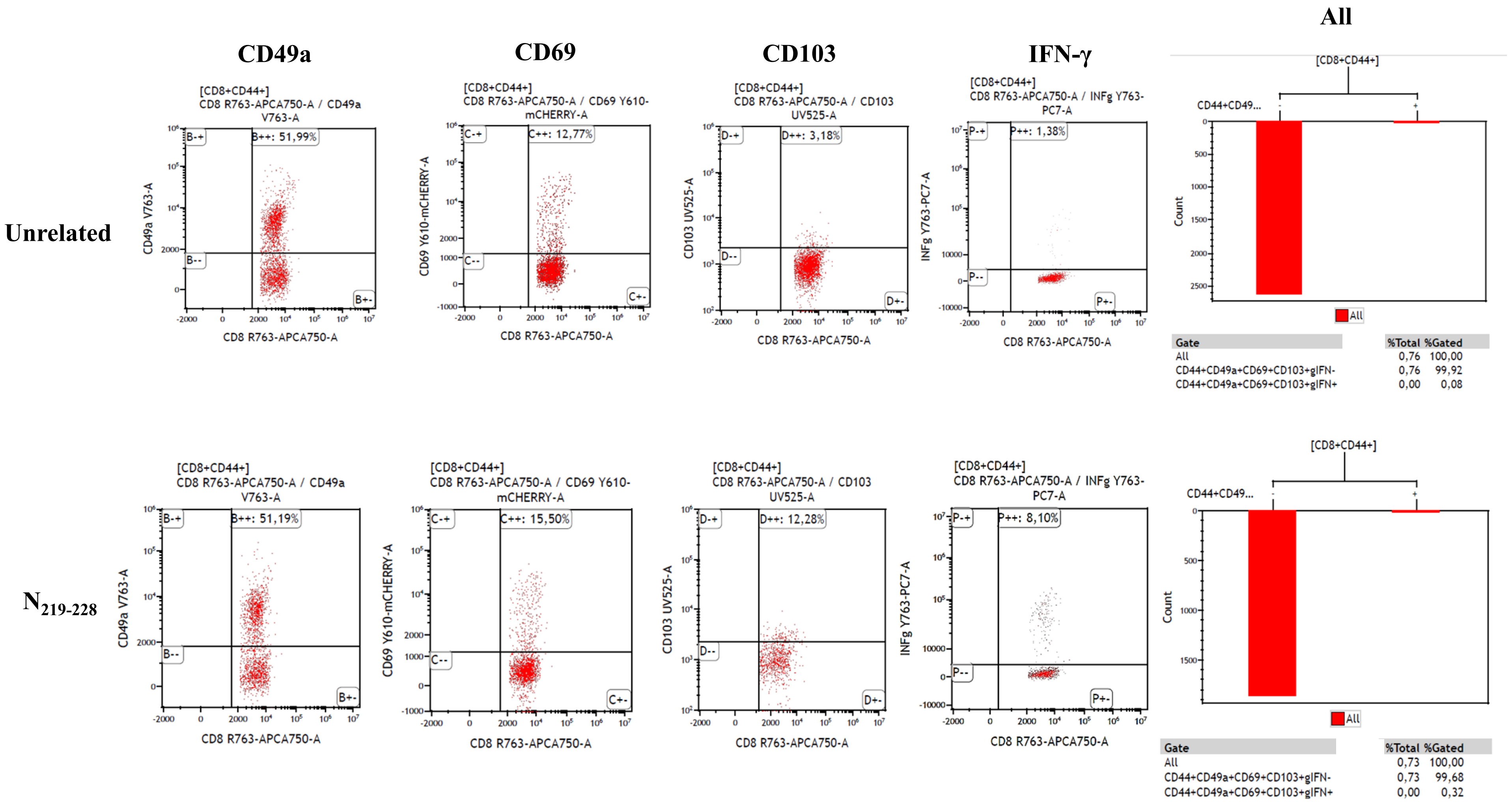

Supplementary Figure S6B. ICS/flow cytometry analysis on cells isolated from lungs of mice immunized with vectors expressing Nef^{mut}/N and injected with anti-CD45 mAbs 3 minutes before sacrifice. Shown are the rough data obtained by analyzing cells pooled from lungs of two representative mice, and treated with either unrelated or N-specific peptides. On the right, Boolean-gating based analysis for the co-expression of all considered markers.
